# Low base‐substitution mutation rate and predominance of insertion‐deletion events in the acidophilic bacterium *Acidobacterium capsulatum*

**DOI:** 10.1002/ece3.8429

**Published:** 2021-12-17

**Authors:** Sibel Kucukyildirim, Samuel F. Miller, Michael Lynch

**Affiliations:** ^1^ Department of Biology Hacettepe University Ankara Turkey; ^2^ Department of Biology Indiana University Bloomington IN USA; ^3^ Biodesign Center for Mechanisms of Evolution Arizona State University Tempe Arizona USA

**Keywords:** acidophile, mutation rate, mutation spectrum

## Abstract

Analyses of spontaneous mutation have shown that total genome‐wide mutation rates are quantitatively similar for most prokaryotic organisms. However, this view is mainly based on organisms that grow best around neutral pH values (6.0–8.0). In particular, the whole‐genome mutation rate has not been determined for an acidophilic organism. Here, we have determined the genome‐wide rate of spontaneous mutation in the acidophilic *Acidobacterium capsulatum* using a direct and unbiased method: a mutation‐accumulation experiment followed by whole‐genome sequencing. Evaluation of 69 mutation accumulation lines of *A*. *capsulatum* after an average of ~2900 cell divisions yielded a base‐substitution mutation rate of 1.22 × 10^−10^ per site per generation or 4 × 10^−4^ per genome per generation, which is significantly lower than the consensus value (2.5−4.6 × 10^−3^) of mesothermophilic (~15–40°C) and neutrophilic (pH 6–8) prokaryotic organisms. However, the insertion‐deletion rate (0.43 × 10^−10^ per site per generation) is high relative to the base‐substitution mutation rate. Organisms with a similar effective population size and a similar expected effect of genetic drift should have similar mutation rates. Because selection operates on the total mutation rate, it is suggested that the relatively high insertion‐deletion rate may be balanced by a low base‐substitution rate in *A*. *capsulatum*, with selection operating on the total mutation rate.

## Graphical abstract

Investigating organisms adapted to life in extreme habitats may further our understanding of the mechanisms of genome stability. Our methods precisely measure the genome‐wide rate, molecular spectrum, and spatial distribution of spontaneous mutations. To our knowledge, this report is the first experimental result of a genome‐wide mutation profile in an acidophilic prokaryote.

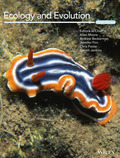

## 1 INTRODUCTION

Spontaneous mutations are the ultimate source of genetic variation and thus a key component of evolution. Under optimal growth conditions, estimates of genome‐wide spontaneous mutation rates in most mesothermophilic (~15–40°C) and neutrophilic (pH 6–8) prokaryotes range from 0.0025 to 0.0046 per genome per generation (Long et al., 2018), despite a wide diversity of life histories and natural habitats. pH, an important environmental factor, has effects on the structure and stability of many biological molecules as well as all biological processes. Although growth patterns of enteric pathogens, such as *Escherichia coli*, *Salmonella* sp., and *Helicobacter pylori* in acidic pH (such as extremely low pH of the stomach during digestion) have been studied extensively (Ansari & Ymaoka, 2017; Ferreira & Lund, 2008; Ramos‐Morales, 2012; Xu et al., 2020), much less is known about the effects of acidic pH on the genome‐wide spontaneous mutation rate in bacteria.

Microorganisms have evolved to grow in different ranges of environmental pH from pH 0 to above pH 13 (Nordstrom et al., 2000; Roadcap et al., 2006; Slonczewski et al., 2009). The external pH has an effect on intracellular pH, which affects all biochemical activities and the structure and stability of both nucleic acids and many other biological molecules. Thus, all biological processes are dependent on pH, for in any cell, the intracellular pH must maintain specific and constant value (within a narrower range than external pH and usually close to neutrality; Slonczewski et al., 2009). For example, under optimal growth conditions (pH = 3.5–3.7 and 75°C), the thermoacidophilic archeon *Sulfolobus acidocaldarius* maintains its intracellular pH around 6.0, with a high pH homeostasis capacity at external acidic pH. Also, acidophilic microorganisms have a number of adaptations to survive in acidic environmental conditions. For example, proteins may have an increased negative surface charge that stabilizes them at low pH (Baker‐Austin & Dopson, 2007; Xia & Palidwor, 2005). Therefore, in order to understand how environmental factors (temperature, pH, etc.) and intrinsic mechanisms (DNA replication and repair) cooperate and determine the genome‐wide mutation rate and spectrum across the tree of life, it is necessary to expand experimental assays to species living in extreme environments.

In this study, we performed a mutation accumulation (MA) experiment combined with whole‐genome sequencing (WGS) on *Acidobacterium capsulatum*, a member of phylum Acidobacteria. The members of the Acidobacteria are abundantly distributed in soil habitats with different physical and biogeochemical characteristics worldwide (Janssen, 2006; Tringe et al., 2005), and can represent 20% of the microbial community across diverse soil environments (Janssen, 2006). Thus, it is assumed that Acidobacteria are genetically and metabolically diverse and, they also have a significant role in biogeochemical processes because of their ubiquity and abundance in various ecosystems (Barns et al., 1999). The first recognized species of Acidobacteria was *A*. *capsulatum*, isolated from an acid‐mine drainage in Japan (Kishimoto et al., 1991). *A*. *capsulatum* grows best at a pH of 3.0–6.0 (optimum 5.0) but does not grow at a pH below 3 or above 6.5. The genome sequence of *A*. *capsulatum* is 4.13 Mb in length with 60.5% GC content (Ward et al., 2009). By expanding previous mutational analyses to an unexplored *A*. *capsulatum* with unusual environmental requirements, this work will enhance the understanding of how different genetic and environmental backgrounds contribute to the mutation process.

## 2 MATERIAL AND METHODS

### 2.1 Mutation accumulation

To estimate the mutation rate in *A*. *capsulatum* (ATCC 51196), 80 independent MA lines were initiated from a single colony of *A*. *capsulatum*. The recommended ATCC agar medium 1168 was used for the mutation‐accumulation line transfers. All lines were incubated at 30°C under aerobic conditions. Every week, a single colony from each line was transferred onto a fresh plate, minimizing the effective population size. This bottlenecking procedure ensures that mutations accumulate in an effectively neutral fashion (Kibota & Lynch, 1996). Every month, single colonies from 10 randomly selected MA lines were used to count colony forming units (CFU) and the mean number of generations (n) was estimated by n = log_2_(CFU). The total number of cell divisions (generations) of each MA line is the product of the mean (18.3) of all generation estimates and the total number of transfers for each line. MA experiment was carried out for ~2900 generations with 69 independent lineages and on average, each MA survived line experienced 159 transfers. Frozen stocks of all lineages were prepared by growing a final colony per isolate in 1 ml ATCC medium 1168 broth medium incubated at 30°C, and frozen in 20% glycerol at −80°C.

### 2.2 DNA extraction, library construction and genome sequencing

MA lines surviving to the end of the MA experiment (69/80) were prepared for WGS. DNA was extracted with the Wizard^®^ Genomic DNA Purification kit (Promega, Madison, Wisconsin, USA). DNA libraries for Illumina HiSeq 2500 sequencing (insert size 300 bp) were constructed using the Nextera DNA Sample Preparation kit (Illumina, San Diego, CA). Paired‐end 150‐nt read sequencing of MA lines was done by the Hubbard Center for Genome Studies, University of New Hampshire.

### 2.3 Mutation analyses

The median depth of coverage for the 69 MA lines was about 139×, and >85% of the genomic sites were covered with reads in all sequenced lines (Supporting Information File S1). All MA lines have at least 20× depth of coverage and no cross‐line contamination. Adaptors of paired‐end reads were removed with Trimmomatic 0.32 (Bolger et al., 2014), and then trimmed reads were mapped to the reference genome (NCBI accession number: NC_012483.1) using BWA 0.7.12 (Li & Durbin, 2009). Then, SAMtools‐1.3.1 (Li et al., 2009) were used to transform sam files to the bam format. Duplicate reads were removed using picardtools‐1.141 and read realignment around insertion‐deletion using GATK 3.5, before performing SNP and indel discovery with standard hard filtering parameters described by GATK Best Practices recommendations (except that we set the Phred‐scaled quality score QUAL > 100 and RMS mapping quality MQ > 59 for both variant and nonvariant sites) (DePristo et al., 2011; McKenna et al., 2010; Van der Auwera et al., 2013) and only unique mutations were included. Base‐pair substitutions and small indels were called using the HaplotypeCaller in GATK. Perl scripts were used to detect variants located in SSRs (https://cci‐git.uncc.edu/wsung/ssrsearch). We also tested the mutation calls by using breseq 0.32.0 (Deatherage & Barrick, 2014). Read alignment for all mutation sites was validated visually with the Integrated Genome Viewer v.2.3.5 (Thorvaldsdottir et al., 2013). Greater than 99% of reads in a line were required to determine the line‐specific consensus nucleotide at a candidate site—a level of 1% was set to allow for aberrant reads originating from sequencing errors, impure indices during library construction, or barcode degeneracy during sequence demultiplexing.

### 2.4 Calculations and statistics

The pooled mean mutation rate (*μ*) was calculated with the formula $\mu =\frac{m}{\sum_{i=1}^{n}N_{i}\times T_{i}}$, where *m* is the total number of observed mutations across all MA lines, *n* is the total number of MA lines, *N_i_* is the number of sites analyzed in each MA line, and *T_i_* is the number of generations for the line. The standard error of the mean mutation rate was calculated with the equation $SEM=\frac{\mathrm{SD}}{\surd n}$, where *SD* is the standard deviation of mutation rates of each line. The expected GC content at mutation equilibrium was calculated with the formula: $\frac{\mu A/T\to G/C}{\mu G/C\to A/T+\mu A/T\to G/C}$, where μA/T→G/C is the mutation rate in the GC direction (the sum of the A/T → G/C transition rate and the A/T → C/G transversion rate), and μG/C→A/T is the mutation rate in the AT direction (the sum of the G/C → A/T transition rate and the G/C → T/A transversion rate) (Lynch, 2007).

All statistical tests and plotting were performed in R v3.1.0 (R Development Core Team, 2014); 95% Poisson confidence intervals of mutation‐rate estimates were calculated using cumulative distribution function approximated by the *χ* ^2^ distribution in R. Mutation analyses were done using the Karst computation cluster of Indiana University.

## 3 RESULTS

### 3.1 Base‐substitution mutations

Across the 69 sequenced *A*. *capsulatum* MA lines (with an average of 3.5 Mb analyzable sequence per line, ~86% of the total genome), we identified 87 base‐substitution mutations, yielding an overall base‐substitution mutation rate of 1.22 × 10^−10^ (95% confidence intervals: 0.98 × 10^−10^, 1.51 × 10^−10^) per nucleotide site per generation, or 0.0004 (*SE* = 0.00005) per genome per generation (Table S1). The number of base‐substitution mutations detected by the breseq pipeline was 97, and 84.5% of these mutations were also identified with the GATK method (Table S2). The base‐pair substitution mutation rate from the breseq method is 1.36 × 10^−10^ per site per generation (95% confidence intervals: 1.10 × 10^−10^, 1.67 × 10^−10^), which is not significantly different than the value calculated using GATK.

Using the annotated *A*. *capsulatum* genome (NCBI accession: NC_012483.1), we determined that63 of the 87 (72.4%) substitutions are in coding regions, while the remaining 24 are found at noncoding sites (TableS3), consistent with the overall coding percentage (88.3% of the genome represents coding regions). We found that the base‐substitution mutation rate of *A*. *capsulatum* in coding regions (1.00 × 10^−10^ per nucleotide site per generation; 95% confidence intervals: 0.77 × 10^−10^, 1.28 × 10^−10^) and noncoding regions (2.32 × 10^−10^; 95% confidence intervals: 1.49 × 10^−10^, 3.46 × 10^−10^), are significantly different (Fisher's exact test, *p* < .05). Among base‐substitutions in coding regions, 25 of 63 (39.7%) are synonymous. We then asked whether the ratio of nonsynonymous to synonymous mutations is significantly different from the random expectation. Given the codon usage and the transition/transversion ratio in *A. capsulatum*, the mutations is 2.76, which is not different from the observed ratio of 2.12 (*χ* ^2^ = 1.09, *df* = 1, *p* = .29). Thus, selection does not appear to have had a significant influence on the distribution of mutations in this experiment.

We found 57 transitions and 31 transversions, resulting in a transition/transversion ratio of 1.84. Among the base‐substitution changes, there are 40 G:C → A:T transitions and 10 G:C → T:A transversions at GC sites, yielding a mutation rate in the AT direction of $\mu_{G/C\to A/T}$ = 1.16 × 10^−10^ per site per generation. In contrast, 17 A:T → G:C transitions and 3 A:T → C:G transversions yielded a mutation rate in the G:C direction of $\mu_{A/T\to G/C}$ = 0.71 × 10^−10^ per site per generation (Table S1), which is lower than the $\mu_{G/C\to A/T}$ rate (95% Poisson confidence intervals for $\mu_{G/C\to A/T}$ 0.86−1.53 × 10^−10^, for $\mu_{A/T\to G/C}$ 0.43−1.1 × 10^−10^). Given these conditional A/T↔G/C mutation rates, the expected genomic GC content if mutation alone is the driving process is 38%, significantly lower than the actual chromosomal GC content of 60.5%. Our results are consistent with a hypothesis of near‐universal mutation bias toward A/T countered by selection for GC content (Hershberg & Petrov, 2010; Long et al., 2018).

### 3.2 Small insertions and deletions

We identified 31 short insertions and deletions, 1–50 bps in length, yielding an insertion‐deletion rate of 4.35 × 10^−11^ (95% confidence intervals: 2.96 × 10^−11^, 6.18 × 10^−11^) per site per generation (Tables [Link], [Link], [Link], [Link]). The number of insertion and deletions detected by the breseq method was 45 and about 65% of these mutations were the same with the GATK method (Table S2). The indel mutation rate from the breseq method is 6.32 × 10^−11^ per site per generation (95% confidence intervals: 4.61 × 10^−11^, 8.45 × 10^−11^), which is not significantly different than the value calculated using GATK. 20 of the 31 (64.5%) indels are in coding regions (3.18 × 10^−11^ per nucleotide site per generation; 95% confidence intervals: 1.94 × 10^−11^, 4.91 × 10^−11^), while the remaining 11 are found at noncoding sites (1.06 × 10^−10^ per nucleotide site per generation; 95% confidence intervals: 0.53 × 10^−10^, 1.91 × 10^−11^; Table S4). Our analysis also showed that indels are not randomly distributed through genome and are underrepresented in protein‐coding regions (observed: 20, expected: 27; Fisher's exact test, *p* > .05) and overrepresented in noncoding regions (observed: 11, expected: 4; Fisher's exact test, *p* < .05).

We found 15 insertions and 16 deletions, implying that the insertion rate (2.10 × 10^−11^ per site per generation; 95% confidence intervals: 1.16 × 10^−11^, 3.42 × 10^−11^) is not different than the deletion rate (2.11 × 10^−11^ per site per generation; 95% confidence intervals: 1.27 × 10^−11^, 3.59 × 10^−11^). However, the total size of all insertions is 90 bp while the deletions total 143 bp, resulting in a net loss of 53 bp in DNA sequence across all lines, consistent with the near universal prokaryotic deletion bias hypothesis (Mira et al., 2001).

**FIGURE 1:**
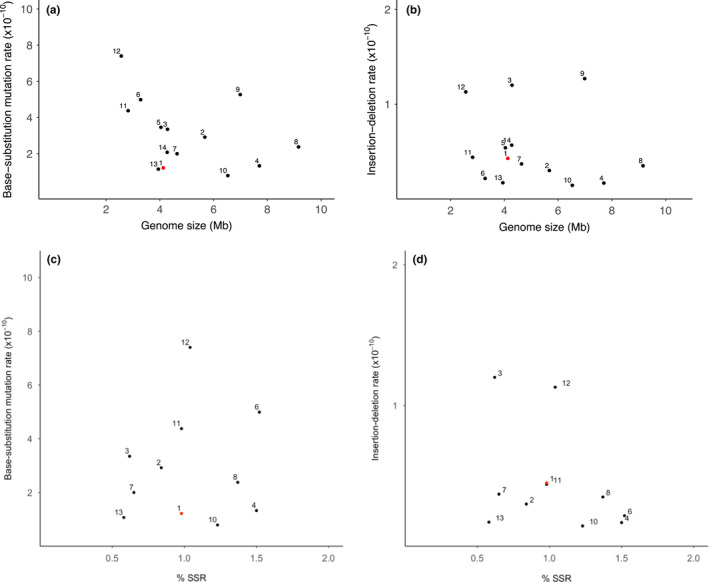
(a) (Base‐substitution mutation rate) and (b) (insertion‐deletion rate) show the relationships between the mutation rate (/site/generation) and total haploid genome size. (c) (Base‐substitution mutation rate) and (d) (insertion‐deletion rate) show the relationships between the mutation rate and abundance of simple sequence repeats within the genome (% SSR). Data points correspond to the following species: 1: *A*. *capsulatum*, 2: *Agrobacterium tumefaciens*, 3: *Bacillus subtilis*, 4: *Bulkholderia cenocepacia*; 5: *Caulobacter crescentus*, 6: *Deinococcus radiodurans*, 7: *E*. *coli*, 8: *Gemmata obscuriglobus*, 9: *Mycobacterium smegmatis*, 10: *Pseudomonas aeruginosa*, 11: *S*. *aureus*, 12: *Staphylococcus epidermidis*, 13: *Vibrio cholera*, 14: *Vibrio fischeri* (data derived from Dettman et al., 2016; Long et al., 2018; Sung et al., 2016)

Though *A*. *capsulatum* has a low base‐substitution mutation rate, its indel rate is higher than previous analyses of genome‐wide spontaneous mutations in prokaryotes (Long et al., 2018; Sung et al., 2016). While indel mutations range from 1.8% to 11.9% of total mutations in most organisms (Sung et al., 2016), we found that >26% of total mutations are indel mutations in *A*. *capsulatum*. To understand the high indel rate observed in this organism, we further examined the simple sequence repeat regions (SSRs), which are well‐known as mutational hotspots. The *A*. *capsulatum* genome has 2474 SSRs, located mainly in coding regions (92.4%). These regions cover 0.98% of the genome, similar to that found in other prokaryotic genomes (Mrazek et al., 2007). We found that 38.7% (12/31) of the small indels occur in SSRs in *A*. *capsulatum* (Table S4), and that 8 of the 12 indel mutations are both in SSRs and coding regions. We then surveyed bacterial genomes by focusing on the relationship between SSR abundance and the indel rate in bacteria. However, we could not find such a relationship, except that the *A*. *capsulatum* indel rate is not different than that of *Staphylococcus aureus* with the same SSR percentage (Figure 1).

## 4 DISCUSSION

Accurate estimates of mutation rates and spectra across organisms adapted to life in extreme habitats are necessary for a comprehensive view of evolution. Here, we report measures of the genome‐wide rate, spectrum, and distribution of spontaneous mutations in the acidophilic bacterium *A*. *capsulatum*. Previous estimates of genome‐wide spontaneous mutations in prokaryotes under optimal growth conditions have shown that most mesothermophilic (~15–40°C) and neutrophilic (pH 6–8) bacteria share two mutational characteristics. First, they have remarkably similar genome‐wide base‐substitution mutation rates ranging from 0.0025 to 0.0046 per genome per generation (Drake, 1991; Long et al., 2018; Lynch et al., 2016; Strauss et al., 2017). Second, insertion‐deletion mutations range from 1.8% to 11.9% of total mutations in an organism (Sung et al., 2016). Interestingly, we found a low genome‐wide base‐substitution rate of 0.0004, compared to rates estimated in other neutrophilic (that commonly need pH 6–8 for optimum activity) prokaryotes, and a relatively high indel rate compared to the base‐substitution mutations. The observed low base‐substitution rate suggests that either *A*. *capsulatum* replication fidelity is higher than in other prokaryotes or alternative biochemical repair mechanisms are used to maintain a low mutation rate. But, the relatively high indel rate may be a consequence of the underlying molecular mechanisms that arise and repair base‐substitution and indels (Kunkel, 2009; Sung et al., 2015). While indels mainly derive from strand slippage or double‐strand breaks, and are often repaired by nucleotide‐excision repair, base‐substitutions mostly result from base misincorporation or biochemical alteration, and are primarily reversed by alkyl transferases or base‐excision repair (Morita et al., 2010).

According to the drift barrier hypothesis (Lynch et al., 2016; Sung et al., 2012), the efficacy of selection in reducing the mutation rate is determined by the power of random genetic drift, which is inversely proportional to the effective population size. Thus, it is expected different that bacterial species have roughly similar per genome mutation rates if they have similar effective population sizes. Consistent with this view, it is possible that the relatively low base substitution mutation rate in *A*. *capsulatum* is balanced by a relatively high insertion‐deletion rate, as selection operates primarily on the total genome‐wide mutation rate, and less so on the detailed molecular spectrum of mutations (Lynch et al., 2016; Sung et al., 2012, 2016; Figure 1). While this work contributes to our understanding of mutation rates and spectra, and how these factors may differ among organisms, further studies with other extremophiles will help provide a deeper understanding of how mutational processes are shaped by intrinsic and extrinsic conditions.

## CONFLICTS OF INTEREST

No conflicts of interest.

## AUTHOR CONTRIBUTIONS

**Sibel Kucukyildirim:** Data curation (lead); formal analysis (lead); investigation (equal); writing – original draft (lead); writing – review & editing (lead). **Samuel F. Miller:** Investigation (equal); methodology (equal); writing – review & editing (supporting). **Michael Lynch:** Conceptualization (lead); funding acquisition (lead); project administration (lead); writing – review & editing (supporting).

## Supporting information

Table S1

Table S2

Table S3

Table S4

## Data Availability

Raw Illumina sequence data reported in this study has been deposited in NCBI SRA (Bioproject No.: PRJNA667213) and Dryad (https://doi.org/10.5061/dryad.rr4xgxd9b).
