# Supplementary material for: Low base‐substitution mutation rate and predominance of insertion‐deletion events in the acidophilic bacterium Acidobacterium capsulatum
Source: Ecol Evol. 2021 Dec 17;11(24):17609–14. doi: 10.1002/ece3.8429 (PMC8717266; doi:10.1002/ece3.8429)
Supplement: Supplementary file 1 — Table S1 [file ECE3-11-17609-s001.pdf]

Suppl. Table 1. ***Acidobacterium capsulatum*** **genome-wide base substitution and inserion-deletion (indel) summary statistics**

MA Line	Cov*	A sites	C sites	G sites	T sites	Substitutions												Indels				Total sites	Gen.	Base-sub Rate	Indel Rate
						Transitions				Transversions								Ins.		Del.					
						GC>AT		AT>GC		AT>TA		GC>TA		AT>CG		GC>CG									
Ac_1	235	704390	1076931	1065185	698744	0	0	0	0	0	0	0	0	0	0	0	0	1	9.63E-11	0	0	3545250	2928	0	9.63E-11
Ac_2	57	704404	1076950	1065204	698754	0	0	0	0	0	0	0	0	0	0	0	0	0	0	0	0	3545312	2928	0	0
Ac_3	107	704404	1076950	1065204	698754	0	0	0	0	0	0	0	0	0	0	0	0	0	0	0	0	3545312	2928	0	0
Ac_5	217	704404	1076950	1065204	698754	2	3.21E-10	0	0	0	0	0	0	0	0	0	0	0	0	0	0	3545312	2910	1.94E-10	0
Ac_6	147	704404	1076950	1065204	698754	2	3.21E-10	0	0	0	0	0	0	0	0	2	3.21E-10	1	9.69E-11	0	0	3545312	2910	3.88E-10	9.69E-11
Ac_7	168	704404	1076950	1065204	698754	0	0	0	0	0	0	0	0	0	0	1	1.59E-10	0	0	0	0	3545312	2928	9.63E-11	0
Ac_8	178	704404	1076950	1065204	698754	0	0	0	0	1	2.43E-10	0	0	0	0	0	0	1	9.63E-11	0	0	3545312	2928	9.63E-11	9.63E-11
Ac_9	134	704404	1076950	1065203	698754	1	1.6E-10	1	2.45E-10	0	0	0	0	0	0	0	0	0	0	1	9.69E-11	3545311	2910	1.94E-10	9.69E-11
Ac_10	106	704404	1076950	1065204	698754	0	0	0	0	0	0	0	0	0	0	0	0	0	0	0	0	3545312	2910	0	0
Ac_11	137	704404	1076950	1065204	698754	0	0	0	0	0	0	2	3.19E-10	0	0	0	0	0	0	1	9.63E-11	3545312	2928	1.93E-10	9.63E-11
Ac_12	89	704404	1076950	1065203	698754	0	0	1	2.43E-10	0	0	0	0	0	0	0	0	0	0	1	9.63E-11	3545311	2928	9.63E-11	9.63E-11
Ac_13	93	704402	1076947	1065201	698754	2	3.19E-10	0	0	0	0	0	0	0	0	1	1.59E-10	1	9.63E-11	0	0	3545304	2928	2.89E-10	9.63E-11
Ac_14	96	704404	1076950	1065204	698754	0	0	0	0	0	0	0	0	0	0	0	0	0	0	0	0	3545312	2928	0	0
Ac_15	101	704404	1076950	1065204	698754	1	1.59E-10	3	7.3E-10	0	0	0	0	0	0	0	0	0	0	0	0	3545312	2928	3.85E-10	0
Ac_16	106	704404	1076950	1065204	698754	1	1.59E-10	0	0	0	0	0	0	0	0	0	0	0	0	0	0	3545312	2928	9.63E-11	0
Ac_17	247	704404	1076950	1065204	698754	2	3.19E-10	0	0	0	0	0	0	0	0	0	0	0	0	0	0	3545312	2928	1.93E-10	0
Ac_18	161	704404	1076950	1065204	698754	1	1.59E-10	0	0	0	0	0	0	0	0	0	0	0	0	0	0	3545312	2928	9.63E-11	0
Ac_19	150	704404	1076948	1065202	698752	0	0	2	4.9E-10	0	0	0	0	0	0	0	0	0	0	1	9.69E-11	3545306	2910	1.94E-10	9.69E-11
Ac_20	112	704404	1076950	1065204	698754	0	0	0	0	1	2.45E-10	0	0	0	0	0	0	1	9.69E-11	0	0	3545312	2910	9.69E-11	9.69E-11
Ac_23	90	704391	1076935	1065190	698741	0	0	0	0	0	0	0	0	0	0	0	0	0	0	1	9.69E-11	3545257	2910	0	9.69E-11
Ac_24	144	704382	1076922	1065157	698725	4	6.42E-10	1	2.45E-10	0	0	0	0	0	0	0	0	0	0	0	0	3545186	2910	4.85E-10	0
Ac_25	233	704404	1076950	1065204	698754	0	0	0	0	0	0	0	0	0	0	0	0	0	0	0	0	3545312	2910	0	0
Ac_26	192	704404	1076950	1065204	698754	0	0	0	0	0	0	0	0	0	0	0	0	0	0	0	0	3545312	2910	0	0
Ac_27	112	704404	1076950	1065204	698754	0	0	0	0	0	0	0	0	0	0	0	0	0	0	0	0	3545312	2910	0	0
Ac_28	178	704404	1076950	1065204	698754	1	1.6E-10	0	0	0	0	0	0	0	0	0	0	0	0	0	0	3545312	2910	9.69E-11	0
Ac_29	210	704404	1076950	1065204	698754	0	0	0	0	0	0	0	0	0	0	0	0	0	0	0	0	3545312	2928	0	0
Ac_30	73	704404	1076950	1065204	698754	1	1.59E-10	0	0	0	0	0	0	0	0	0	0	0	0	0	0	3545312	2928	9.63E-11	0
Ac_31	227	704404	1076950	1065204	698754	1	1.59E-10	0	0	0	0	0	0	0	0	0	0	0	0	0	0	3545312	2928	9.63E-11	0
Ac_33	182	704404	1076950	1065204	698754	0	0	0	0	0	0	0	0	0	0	0	0	0	0	0	0	3545312	2910	0	0
Ac_34	197	704402	1076948	1065203	698754	1	1.6E-10	0	0	0	0	0	0	0	0	0	0	1	9.69E-11	0	0	3545307	2910	9.69E-11	9.69E-11
Ac_35	226	704404	1076950	1065204	698754	1	1.59E-10	0	0	1	2.43E-10	0	0	0	0	0	0	0	0	0	0	3545312	2928	1.93E-10	0
Ac_36	215	704404	1076950	1065204	698754	1	1.59E-10	0	0	0	0	0	0	0	0	0	0	1	9.63E-11	0	0	3545312	2928	9.63E-11	9.63E-11
Ac_37	226	704404	1076950	1065204	698754	0	0	0	0	0	0	0	0	0	0	0	0	0	0	0	0	3545312	2910	0	0
Ac_39	155	704404	1076950	1065204	698754	0	0	0	0	0	0	0	0	0	0	0	0	0	0	0	0	3545312	2910	0	0
Ac_40	228	704404	1076950	1065204	698754	0	0	0	0	0	0	0	0	0	0	0	0	0	0	0	0	3545312	2910	0	0
Ac_41	156	704404	1076950	1065204	698754	0	0	0	0	0	0	0	0	0	0	0	0	0	0	0	0	3545312	2928	0	0
Ac_42	219	704404	1076950	1065204	698754	0	0	0	0	1	2.43E-10	0	0	0	0	0	0	0	0	0	0	3545312	2928	9.63E-11	0
Ac_43	210	704404	1076950	1065204	698754	0	0	0	0	0	0	1	1.61E-10	0	0	0	0	0	0	0	0	3545312	2891	9.76E-11	0

Ac_44	229	704404	1076950	1065204	698754	0	0	0	0	0	0	0	0	0	0	0	0	0	0	0	0	3545312	2891	0	0
Ac_45	189	704404	1076950	1065204	698754	0	0	1	2.46E-10	0	0	0	0	0	0	0	0	0	0	1	9.76E-11	3545312	2891	9.76E-11	9.76E-11
Ac_46	142	704404	1076949	1065203	698752	0	0	0	0	0	0	0	0	0	0	0	0	0	0	1	9.76E-11	3545308	2891	0	9.76E-11
Ac_47	229	704404	1076950	1065204	698754	0	0	1	2.46E-10	0	0	1	1.61E-10	0	0	0	0	0	0	1	9.76E-11	3545312	2891	1.95E-10	9.76E-11
Ac_48	226	704404	1076950	1065204	698754	1	1.61E-10	0	0	0	0	0	0	0	0	0	0	0	0	0	0	3545312	2891	9.76E-11	0
Ac_49	198	704404	1076950	1065204	698754	0	0	0	0	0	0	0	0	0	0	0	0	1	9.82E-11	0	0	3545312	2873	0	9.82E-11
Ac_50	180	704404	1076950	1065204	698754	1	1.62E-10	0	0	1	2.48E-10	0	0	0	0	0	0	0	0	0	0	3545312	2873	1.96E-10	0
Ac_52	158	704404	1076950	1065204	698754	0	0	1	2.46E-10	0	0	1	1.61E-10	0	0	0	0	0	0	0	0	3545312	2891	1.95E-10	0
Ac_53	141	704404	1076950	1065204	698754	3	4.84E-10	0	0	0	0	0	0	0	0	0	0	0	0	0	0	3545312	2891	2.93E-10	0
Ac_54	52	704404	1076950	1065204	698754	0	0	0	0	0	0	1	1.61E-10	0	0	0	0	0	0	0	0	3545312	2891	9.76E-11	0
Ac_55	86	704404	1076950	1065204	698754	0	0	0	0	0	0	0	0	1	2.4493E-10	0	0	0	0	0	0	3545312	2910	9.69E-11	0
Ac_56	82	704404	1076950	1065204	698754	0	0	0	0	0	0	0	0	0	0	1	1.6E-10	0	0	0	0	3545312	2910	9.69E-11	0
Ac_57	93	704404	1076950	1065204	698754	0	0	0	0	0	0	0	0	0	0	0	0	0	0	0	0	3545312	2891	0	0
Ac_58	111	704387	1076926	1065171	698734	0	0	0	0	0	0	0	0	0	0	0	0	0	0	0	0	3545218	2891	0	0
Ac_59	224	704404	1076950	1065204	698754	1	1.59E-10	0	0	0	0	0	0	0	0	1	1.59E-10	0	0	0	0	3545312	2928	1.93E-10	0
Ac_60	116	704383	1076918	1065175	698724	2	3.19E-10	1	2.43E-10	0	0	0	0	0	0	2	3.19E-10	1	9.63E-11	0	0	3545200	2928	4.82E-10	9.63E-11
Ac_61	104	704404	1076950	1065204	698754	0	0	0	0	1	2.45E-10	1	1.6E-10	1	2.4493E-10	0	0	1	9.69E-11	0	0	3545312	2910	2.91E-10	9.69E-11
Ac_62	95	704404	1076949	1065203	698754	1	1.6E-10	1	2.45E-10	0	0	1	1.6E-10	0	0	0	0	0	0	2	1.94E-10	3545310	2910	2.91E-10	1.94E-10
Ac_63	66	704404	1076950	1065204	698754	0	0	0	0	0	0	1	1.61E-10	0	0	0	0	0	0	0	0	3545312	2891	9.76E-11	0
Ac_65	78	704404	1076950	1065204	698754	2	3.21E-10	0	0	0	0	0	0	0	0	0	0	0	0	0	0	3545312	2910	1.94E-10	0
Ac_66	72	704404	1076950	1065204	698754	0	0	0	0	1	2.45E-10	0	0	0	0	0	0	0	0	0	0	3545312	2910	9.69E-11	0
Ac_67	46	704404	1076950	1065204	698754	1	1.6E-10	1	2.45E-10	0	0	0	0	0	0	0	0	1	9.69E-11	1	9.69E-11	3545312	2910	1.94E-10	1.94E-10
Ac_68	49	704401	1076949	1065198	698753	0	0	0	0	0	0	0	0	0	0	0	0	0	0	2	1.94E-10	3545301	2910	0	1.94E-10
Ac_69	54	704404	1076950	1065204	698754	2	3.19E-10	0	0	0	0	0	0	0	0	0	0	1	9.63E-11	0	0	3545312	2928	1.93E-10	9.63E-11
Ac_74	26	704404	1076949	1065200	698754	1	1.61E-10	0	0	1	2.46E-10	0	0	0	0	0	0	1	9.76E-11	0	0	3545307	2891	1.95E-10	9.76E-11
Ac_75	65	704404	1076950	1065204	698754	1	1.6E-10	0	0	0	0	0	0	0	0	0	0	0	0	1	9.69E-11	3545312	2910	9.69E-11	9.69E-11
Ac_76	51	704404	1076950	1065204	698754	0	0	2	4.9E-10	0	0	0	0	0	0	0	0	0	0	0	0	3545312	2910	1.94E-10	0
Ac_77	71	704404	1076950	1065204	698754	0	0	0	0	0	0	0	0	0	0	1	1.59E-10	0	0	0	0	3545312	2928	9.63E-11	0
Ac_78	130	704404	1076950	1065204	698754	1	1.59E-10	0	0	0	0	0	0	1	2.434E-10	0	0	1	9.63E-11	0	0	3545312	2928	1.93E-10	9.63E-11
Ac_79	57	704404	1076950	1065204	698753	1	1.59E-10	0	0	0	0	0	0	0	0	0	0	0	0	0	0	3545311	2928	9.63E-11	0
Ac_80	35	704402	1076946	1065200	698751	0	0	1	2.43E-10	0	0	1	1.59E-10	0	0	0	0	1	9.63E-11	2	1.93E-10	3545299	2928	1.93E-10	2.89E-10
	139					40		17		8		10		3		9		15		16					
**Overall mut rate**							1.42E-10		3.95E-11		1.86E-11		3.55E-11		6.9691E-12		3.19E-11		2.11E-11		2.25E-11	3545305	2912	1.22E-10	4.35E-11
							6.54E-11		3.46E-11		1.21E-11		1.26E-11		2.16E-12		9.42E-12		1.16E-11		1.27E-11			9.78E-11	2.96E-11
							1.25E-10		9.51E-11		5.51E-11		4.51E-11		3.06E-11		3.91E-11		3.42E-11		3.59E-11			1.51E-10	6.18E-11
**SEM****							1.62E-11		1.69E-11		9.44E-12		7.55E-12		6.0013E-12		7.97E-12		4.8E-12		6E-12			1.41E-11	7.56E-12

* Cov is the coverage.

** SEM is the standart error.
