# Supplementary material for: Low base‐substitution mutation rate and predominance of insertion‐deletion events in the acidophilic bacterium Acidobacterium capsulatum
Source: Ecol Evol. 2021 Dec 17;11(24):17609–14. doi: 10.1002/ece3.8429 (PMC8717266; doi:10.1002/ece3.8429)
Supplement: Supplementary file 2 — Table S2 [file ECE3-11-17609-s004.pdf]

Suppl. Table 2. ***Acidobacterium capsulatum*** **genome-wide base substitution and inserion-deletion (indel) predicted by breseq pipeline**

Position	Mutation	MA LINE																																																																					Annotation	Gene	Description
		10	11	12	13	14	15	16	17	18	19	1	20	23	24	25	26	27	28	29	2	30	31	33	34	35	36	37	39	3	40	41	42	43	44	45	46	47	48	49	50	52	53	54	55	56	57	58	59	5	60	61	62	63	65	66	67	68	69	6	74	75	76	77	78	79	7	80	8	9			
3.96	Δ1 bp			+																																	+																																				coding (518/768 nt)	*ACP_RS00025* ←	hypothetical protein
4.371	(GTAAATA)$_{1\rightarrow 2}$																																																						+				+											coding (107/768 nt)	*ACP_RS00025* ←	hypothetical protein
29.743	A→G												+																																																										T875A (ACG→GCG)	*ACP_RS00115* →	TonB-dependent receptor
48.001	T→C																																																							+														T116G (GGT→GGC)	*ACP_RS00175* →	amino acid deaminase
71.983	G→A																								+																																																	V56M (GTG→ATG)	*ACP_RS00255* →	glycoside hydrolase family 27 protein
173.318	G→C				+																																																																						V85V (GTG→GTC)	*ACP_RS00640* →	acetyltransferase
288.009	Δ12 bp																																																																				+			coding (192-203/498 nt)	*flgC* →	flagellar basal body rod protein FlgC
334.628	G→T		+																																																																							intergenic (+166/+289)	*ACP_RS01400* → / ← *ACP_RS01405*	ATP synthase F0 subunit C/NADH-quinone oxidoreductase subunit N
380.915	(G)$_{6\rightarrow 7}$																	+																																																							intergenic (-321/+302)	*ACP_RS01595* ← / ← *ACP_RS01605*	methylmalonyl-CoA mutase family protein/hypothetical protein
389.474	C→G																																																											+											T360T (ACG→ACC)	*ACP_RS01640* ←	aspartyl protease family protein
397.727	G→C																					+																																																					*549Y (TAG→TAC)	*ACP_RS01670* →	cryptochrome/photolyase family protein
401.411	(C)$_{6\rightarrow 5}$		+																																																																								coding (1093/1212 nt)	*ACP_RS01690* ←	RHS repeat-associated core domain-containing protein
431.174	C→T																					+																																																					A33T (GCT→ACT)	*ACP_RS01840* ←	hypothetical protein
448.904	G→C																																																											+											L156L (CTG→CTC)	*ACP_RS17080* →	hypothetical protein
482.088	Δ11 bp																																																									+													coding (610-620/993 nt)	*ACP_RS02120* ←	4Fe-4S dicluster domain-containing protein
515.398	T→C																																			+																																							T277T (ACT→ACC)	*ACP_RS02270* →	hypothetical protein
551.031	T→C																																																									+													Q88R (CAG→CGG)	*ACP_RS02405* ←	response regulator
562.22	G→A																																									+																													H299Y (CAT→TAT)	*tolB* ←	Tol-Pal system beta propeller repeat protein TolB
578.388	C→T																																																											+											E292E (GAG→GAA)	*ftcD* ←	glutamate formimidoyltransferase
589.956	G→A																																						+																																			F4F (TTC→TTT)	*ruvC* ←	crossover junction endodeoxyribonuclease RuvC
590.922	T→G									+																																																															T193P (ACC→CCC)	*ACP_RS02580* ←	Tat pathway signal sequence domain-containing protein
670.329	G→A																																																					+																		G104D (GGC→GAC)	*ACP_RS02835* →	VOC family protein
673.065	G→A																																																												+										R579C (CGC→TGC)	*ACP_RS02855* ←	glycine--tRNA ligase subunit beta

722.614	T→C	+																					H102H (CAT→CAC)	*ACP_RS03065* →	metallophosphoesterase family protein
758.498	$(G)_{7\rightarrow8}$																				+		intergenic (+367/+62)	*ACP_RS03215* → / ← *ACP_RS03220*	GlxA family transcriptional regulator/thioredoxin domain-containing protein
759.257	A→T						+	+	+		+	+											V103D (GTC→GAC)	*ACP_RS03220* ←	thioredoxin domain-containing protein
787.294	T→C																		+				T46A (ACG→GCG)	*ACP_RS03330* ←	glycosyltransferase family 2 protein
787.298	3 bp→A																		+				coding (130-132/1203 nt)	*ACP_RS03330* ←	glycosyltransferase family 2 protein
798.573	G→T														+								intergenic (-487/-613)	*ACP_RS03365* ← / → *ACP_RS17145*	pesticidal protein Cry15Aa/EAL domain-containing protein
834.952	T→A													+									L70Q (CTG→CAG)	*ACP_RS03515* →	formate--tetrahydrofolate ligase
850.658	G→A		+																				S246S (AGC→AGT)	*hldE* ←	bifunctional D-glycero-beta-D-manno-heptose-7-phosphate kinase/D-glycero-beta-D-manno-heptose 1-phosphate adenylyltransferase HldE
881.692	G→A									+													intergenic (-51/+73)	*ACP_RS03700* ← / ← *ACP_RS03705*	DUF4126 family protein/TetR/AcrR family transcriptional regulator
894.402	Δ133 bp			+																		?	intergenic (+525/+1350)	*ACP_RS03730* → / ← *cas2*	discoidin domain-containing protein/CRISPR-associated endonuclease Cas2
894.468	Δ132 bp																+						intergenic (+591/+1285)	*ACP_RS03730* → / ← *cas2*	discoidin domain-containing protein/CRISPR-associated endonuclease Cas2
895.128	Δ197 bp					+																	intergenic (+1251/+560)	*ACP_RS03730* → / ← *cas2*	discoidin domain-containing protein/CRISPR-associated endonuclease Cas2
914.651	G→A															+							A139V (GCA→GTA)	*ACP_RS03815* ←	uracil-DNA glycosylase
985.254	C→A											+											intergenic (-389/-142)	*ACP_RS04090* ← / → *ACP_RS04095*	APC family permease/alpha-galactosidase
1,005,612	T→A																			+			T169T (ACA→ACT)	*ACP_RS04145* ←	TonB-dependent receptor
1,057,805	$(TGTCAGT)_{4\rightarrow5}$																	+					intergenic (-64/+222)	*ACP_RS04330* ← / ← *ACP_RS04335*	ABC transporter permease/ABC transporter permease
1,070,663	C→T														+								L170L (CTG→CTA)	*mqnE* ←	aminofutalosine synthase MqnE
1,075,056	$(CGG)_{7\rightarrow6}$																				+		coding (1207-1209/1653 nt)	*ACP_RS04385* ←	hypothetical protein
1,136,789	G→A				+		+	+	+		+	+											P6P (CCG→CCA)	*ACP_RS04675* →	YdcF family protein

1,213,414	A→T	+	D167V (GAC→GTC)	*ACP_RS05020* →	response regulator
1,323,304	G→T	+	intergenic (+24/-127)	*ACP_RS05490* → / → *sbnA*	hypothetical protein/2,3-diaminopropionate biosynthesis protein SbnA
1,390,473	C→T	+	intergenic (+537/+114)	*ACP_RS05615* → / ← *ACP_RS05620*	hypothetical protein/PIN domain-containing protein
1,391,567	$(C)_{9\rightarrow8}$	+	intergenic (-17/+202)	*ACP_RS18270* ← / ← *ACP_RS05630*	hypothetical protein/helix-turn-helix transcriptional regulator
1,459,340	A→T	+	E560V (GAG→GTG)	*ACP_RS05920* →	beta-galactosidase
1,470,241	G→A	+	A308A (GCC→GCT)	*ACP_RS05980* ←	serine/threonine protein kinase
1,504,688	$(G)_{5\rightarrow6}$	+	coding (368/1935 nt)	*ACP_RS06080* →	glycoside hydrolase family 127 protein
1,544,837	$(15\text{-bp})_{2\rightarrow1}$	+	intergenic (-88/+185)	*ACP_RS06240* ← / ← *ACP_RS17250*	hypothetical protein/Ig-like domain repeat protein
1,544,851	$(TCCTGCTATCCGGCC)_{2\rightarrow3}$	+ +	intergenic (-102/+185)	*ACP_RS06240* ← / ← *ACP_RS17250*	hypothetical protein/Ig-like domain repeat protein
1,548,382	+G	+	coding (44/3390 nt)	*ACP_RS17250* ←	Ig-like domain repeat protein
1,593,758	Δ13 bp	+	coding (1484-1496/3180 nt)	*ACP_RS06435* →	multidrug efflux RND transporter permease subunit
1,610,135	A→G	+	F196S (TTT→TCT)	*ACP_RS17265* ←	hypothetical protein
1,611,545	$(16\text{-bp})_{2\rightarrow1}$	+	intergenic (-450/+32)	*ACP_RS06485* ← / ← *ACP_RS06490*	PadR family transcriptional regulator/hypothetical protein
1,629,448	G→A	+	intergenic (-157/+68)	*ACP_RS06540* ← / ← *ACP_RS06545*	excisionase family DNA-binding protein/tRNA-Lys
1,663,000	G→A	+	T338I (ACT→ATT)	*ACP_RS17285* ←	putative Ig domain-containing protein
1,663,106	G→A	+	L303F (CTC→TTC)	*ACP_RS17285* ←	putative Ig domain-containing protein
1,760,586	A→C	+	S25A (TCG→GCG)	*ACP_RS07130* ←	hypothetical protein
1,786,696	A→G	+	S19S (AGT→AGC)	*ACP_RS07215* ←	hypothetical protein
1,859,981	C→A	+	intergenic (+211/-125)	*ACP_RS07490* → / → *ACP_RS07495*	Uma2 family endonuclease/AAA family ATPase
1,859,991	2 bp→TG	+	intergenic (+221/-114)	*ACP_RS07490* → / → *ACP_RS07495*	Uma2 family endonuclease/AAA family ATPase
1,887,722	T→A	+	L302H (CTC→CAC)	*ACP_RS07580* →	glycosyl hydrolase family 39

1,914,804	G→A	+	intergenic (+266/+957)	*ACP_RS07665* → / ← *ACP_RS07670*	hypothetical protein/efflux RND transporter periplasmic adaptor subunit
1,938,411	(TCAGTTGTCAGTCC)$_{2\rightarrow3}$	+	intergenic (-101/+269)	*ACP_RS07775* ← / ← *bshC*	hypothetical protein/bacillithiol biosynthesis cysteine-adding enzyme BshC
1,940,044	G→A	+	R75C (CGC→TGC)	*bshC* ←	bacillithiol biosynthesis cysteine-adding enzyme BshC
1,946,783	T→C	+	N90S (AAT→AGT)	*ACP_RS07820* ←	integration host factor subunit beta
1,971,981	G→C	+	D7E (GAC→GAG)	*ACP_RS07930* ←	SocA family protein
1,993,179	(TTGAGGAAGCGG)$_{1\rightarrow2}$	+	coding (2304/3042 nt)	*ACP_RS08025* ←	Rne/Rng family ribonuclease
2,013,771	(GAA)$_{8\rightarrow7}$	+	coding (156-158/747 nt)	*ACP_RS18295* ←	hypothetical protein
2,021,365	(CGCTGACC)$_{3\rightarrow2}$	+	intergenic (+74/+35)	*ACP_RS08140* → / ← *era*	IS481-like element ISAcp2 family transposase/GTPase Era
2,082,223	C→T	+	A214T (GCA→ACA)	*ACP_RS08415* ←	hypothetical protein
2,191,227	(TTTAGCT)$_{2\rightarrow3}$	+	intergenic (+36/-392)	*ACP_RS17355* → / → *ACP_RS08895*	helix-turn-helix domain-containing protein/hypothetical protein
2,272,157	C→T	+	V440I (GTA→ATA)	*ACP_RS09220* ←	hypothetical protein
2,413,858	A→G	+	intergenic (-7/+273)	*ACP_RS09820* ← / ← *ACP_RS09825*	ComF family protein/DUF1284 domain-containing protein
2,456,667	(GTGAACGAGCGGCTGCTCG)$_{1\rightarrow2}$	+	coding (1213/1365 nt)	*gcvPA* →	aminomethyl-transferring glycine dehydrogenase subunit GcvPA
2,484,174	(CAGCAGCGAG)$_{2\rightarrow1}$	+	coding (2316-2325/2667 nt)	*ACP_RS10130* ←	ABC transporter permease
2,514,716	C→G	+	R32G (CGT→GGT)	*ACP_RS10260* →	hypothetical protein
2,533,138	A→G	+	intergenic (-85/+32)	*ACP_RS10320* ← / ← *ACP_RS18470*	DUF2752 domain-containing protein/YXWGXW repeat-containing protein
2,546,888	T→C	+	L212P (CTG→CCG)	*ACP_RS10395* →	alpha/beta hydrolase
2,551,383	G→A	+	intergenic (-41/+5)	*hemB* ← / ← *ACP_RS10430*	porphobilinogen synthase/zinc ribbon domain-containing protein
2,625,754	C→T	+	V75V (GTG→GTA)	*ACP_RS10760* ←	adenylosuccinate synthase
2,632,658	C→T	+	Q121* (CAG→TAG)	*ACP_RS10800* →	carboxypeptidase regulatory-like domain-containing protein

2,633,092	C→A																							+											Y265* (TAC→TAA)	*ACP_RS10800* →	carboxypeptidase regulatory-like domain-containing protein
2,633,137	(T)$_{5\rightarrow 4}$																+	+													+				coding (840/3600 nt)	*ACP_RS10800* →	carboxypeptidase regulatory-like domain-containing protein
2,633,697	+A																								+										coding (1400/3600 nt)	*ACP_RS10800* →	carboxypeptidase regulatory-like domain-containing protein
2,633,943	G→A	+	+	+						+				+					+			+	+				+								W549* (TGG→TAG)	*ACP_RS10800* →	carboxypeptidase regulatory-like domain-containing protein
2,634,017	+A						+													+				+				+						+	coding (1720/3600 nt)	*ACP_RS10800* →	carboxypeptidase regulatory-like domain-containing protein
2,634,113	C→T				+																														Q606* (CAA→TAA)	*ACP_RS10800* →	carboxypeptidase regulatory-like domain-containing protein
2,634,977	(C)$_{5\rightarrow 6}$															+																	+		coding (2680/3600 nt)	*ACP_RS10800* →	carboxypeptidase regulatory-like domain-containing protein
2,635,156	T→G																													+					Y953* (TAT→TAG)	*ACP_RS10800* →	carboxypeptidase regulatory-like domain-containing protein
2,635,299	C→A								+																	+									S1001* (TCA→TAA)	*ACP_RS10800* →	carboxypeptidase regulatory-like domain-containing protein
2,635,372	G→A							+					+																						W1025* (TGG→TGA)	*ACP_RS10800* →	carboxypeptidase regulatory-like domain-containing protein
2,687,364	(GCGCCGAGCGAAT)$_{1\rightarrow 2}$																															+			coding (271/1383 nt)	*ACP_RS11005* ←	L-serine ammonia-lyase
2,707,729	C→T										+																								R28R (CGC→CGT)	*ACP_RS11110* →	FAD-binding oxidoreductase
2,733,537	(CGCTGCTCC)$_{2\rightarrow 1}$											+																							coding (615-623/1650 nt)	*sucB* →	2-oxoglutarate dehydrogenase, E2 component, dihydrolipoamide succinyltransferase
2,747,650	A→G																												+						L374P (CTG→CCG)	*ACP_RS11295* ←	terminase
2,800,149	Δ50 bp					+																													coding (1428-1477/2553 nt)	*ACP_RS11500* →	glucosiduronase
2,824,313	T→C																			+															I34T (ATC→ACC)	*coxB* →	cytochrome c oxidase subunit II
2,844,167	C→G													+																					intergenic (+95/+11)	*ACP_RS11665* → / ← *ACP_RS11670*	CTP synthase/IS481-like element ISAcp2 family transposase

2,864,566	T→C			+																noncoding (1239/1500 nt)	*ACP_RS11765* →	16S ribosomal RNA
2,872,068	G→A														+					F190F (TTC→TTT)	*ACP_RS11800* ←	nitronate monooxygenase
2,880,630	G→T							+												L55L (CTC→CTA)	*ACP_RS11835* ←	putative toxin-antitoxin system toxin component, PIN family
2,899,736	$(CG)_{5\to4}$							+												coding (291-292/1374 nt)	*ACP_RS11905* →	HAMP domain-containing protein
2,899,985	Δ1 bp																	+		coding (540/1374 nt)	*ACP_RS11905* →	HAMP domain-containing protein
2,909,651	+G						+													coding (681/1695 nt)	*ACP_RS11925* ←	trehalase
2,983,187	A→T															+				L268Q (CTG→CAG)	*rlmD* ←	23S rRNA (uracil(1939)-C(5))-methyltransferase RlmD
2,986,081	C→T										+									Q74Q (CAG→CAA)	*ACP_RS12275* ←	biotin--[acetyl-CoA-carboxylase] ligase
3,003,784	$(CCAACTGAGGACTGACAACTGACAACTG)_{1\to2}$								+											intergenic (-283/+5)	*ACP_RS12345* ← / ← *trpD*	SIMPL domain-containing protein/anthranilate phosphoribosyltransferase
3,030,560	C→G									+										intergenic (+242/+11)	*ACP_RS12465* → / ← *ACP_RS12470*	aldo/keto reductase/IS481-like element ISAcp2 family transposase
3,053,098	G→A													+						R562C (CGC→TGC)	*ACP_RS12580* ←	LysM peptidoglycan-binding domain-containing protein
3,068,203	C→A																	+		P405H (CCT→CAT)	*ACP_RS12630* →	ATP-dependent DNA ligase
3,068,321	C→A	+																		S444R (AGC→AGA)	*ACP_RS12630* →	ATP-dependent DNA ligase
3,073,998	C→G				+	+											+			intergenic (+14/+11)	*hslU* → / ← *ACP_RS12660*	ATP-dependent protease ATPase subunit HslU/IS481-like element ISAcp2 family transposase
3,105,194	C→T											+								T142T (ACG→ACA)	*uxaC* ←	glucuronate isomerase
3,136,232	T→C													+						L102P (CTG→CCG)	*ACP_RS12895* →	formate/nitrite transporter family protein
3,142,757	C→T		+																	intergenic (-46/-273)	*ACP_RS12910* ← / → *ACP_RS12915*	response regulator transcription factor/response regulator
3,206,363	Δ1 bp																		+	intergenic (+809/-108)	*ACP_RS13100* → / → *ACP_RS13105*	periplasmic heavy metal sensor/IS481-like element ISAcp2 family transposase
3,213,963	G→A												+							intergenic (+227/+321)	*ACP_RS13125* → / ← *ccsA*	IS481-like element ISAcp2 family transposase/cytochrome c biogenesis protein CcsA

3,213,968	1 bp→GT												+			intergenic (+232/+316)	*ACP_RS13125* → / ← *ccsA*	IS481-like element ISAcp2 family transposase/cytochrome c biogenesis protein CcsA
3,213,972	C→G												+			intergenic (+236/+312)	*ACP_RS13125* → / ← *ccsA*	IS481-like element ISAcp2 family transposase/cytochrome c biogenesis protein CcsA
3,213,987	T→C												+			intergenic (+251/+297)	*ACP_RS13125* → / ← *ccsA*	IS481-like element ISAcp2 family transposase/cytochrome c biogenesis protein CcsA
3,214,015	C→G												+			intergenic (+279/+269)	*ACP_RS13125* → / ← *ccsA*	IS481-like element ISAcp2 family transposase/cytochrome c biogenesis protein CcsA
3,260,823	C→G									+						P100P (CCC→CCG)	*ACP_RS13305* →	LacI family DNA-binding transcriptional regulator
3,265,382	A→G			+												D329D (GAT→GAC)	*ACP_RS13320* ←	TonB-dependent receptor
3,438,364	+G														+	intergenic (-37/-152)	*smpB* ← / → *ACP_RS14080*	SsrA-binding protein SmpB/type II/III secretion system protein
3,453,815	T→C	+														V297A (GTG→GCG)	*ACP_RS14165* →	divalent metal cation transporter
3,527,746	G→T								+							intergenic (+199/+335)	*ACP_RS14475* → / ← *ACP_RS18380*	alkyl hydroperoxide reductase AhpD/hypothetical protein
3,578,274	C→G										+					A81G (GCA→GGA)	*ACP_RS14670* →	mandelate racemase
3,702,647	C→T				+											A344T (GCA→ACA)	*ACP_RS15200* ←	recombinase RecA
3,754,475	C→T						+									R547* (CGA→TGA)	*ACP_RS15445* →	type IV secretion system DNA-binding domain-containing protein
3,764,181	A→G							+								S309P (TCT→CCT)	*ACP_RS15480* ←	ATP-dependent helicase
3,787,699	A→T					+										intergenic (-116/-244)	*ACP_RS15595* ← / → *ACP_RS17865*	helix-turn-helix transcriptional regulator/hypothetical protein
3,795,752	(ACTCCCAATAG)2→1													+		intergenic (-156/+471)	*ACP_RS15630* ← / ← *ACP_RS15635*	excisionase family DNA-binding protein/dihydrodipicolinate synthase family protein
3,805,991	C→T											+				Q782* (CAG→TAG)	*ACP_RS15665* →	TonB-dependent receptor
3,872,314	C→T														+	V10V (GTC→GTT)	*ACP_RS15955* →	efflux RND transporter periplasmic adaptor subunit
3,880,351	C→T		+													A116V (GCC→GTC)	*ACP_RS15970* →	efflux transporter outer membrane subunit

3,975,248	(A)$_{7\rightarrow 8}$	+					intergenic (-521/-332)	*ACP_RS16395* ← / → *ACP_RS17550*	type II toxin-antitoxin system prevent-host-death family antitoxin/thioredoxin family protein
4,074,092	G→A		+				A52V (GCC→GTC)	*ACP_RS16765* ←	DUF3175 domain-containing protein
4,080,731	C→T				+		A88A (GCC→GCT)	*ACP_RS18505* →	hypothetical protein
4,081,306	C→T					+	P156L (CCC→CTC)	*ACP_RS16800* →	serine hydrolase
4,123,155	A→T			+			K387N (AAA→AAT)	*ACP_RS16935* →	dipeptidase
