# Supplementary material for: Low base‐substitution mutation rate and predominance of insertion‐deletion events in the acidophilic bacterium Acidobacterium capsulatum
Source: Ecol Evol. 2021 Dec 17;11(24):17609–14. doi: 10.1002/ece3.8429 (PMC8717266; doi:10.1002/ece3.8429)
Supplement: Supplementary file 3 — Table S3 [file ECE3-11-17609-s003.pdf]

Suppl. Table 3. Base substitution details

MA Line	Scaffolds	Base-substitution	Gene region	Strand	Codon change	Amino acid change	nonsyn/syn	Gene info
Ac_5	NC_012483.1:1940044	G>A	coding	-	CGC>TGC	R>C	nonsyn	hypothetical_protein
Ac_5	NC_012483.1:3805991	C>T	coding	+	CAG>TAG	Q>*	nonsyn	Cna_protein_B-type_domain-containing_protein
Ac_6	NC_012483.1:389474	C>G	coding	-	ACG>ACC	T>T	syn	hypothetical_protein
Ac_6	NC_012483.1:448904	G>C	coding	+	CTG>CTC	L>L	syn	hypothetical_protein
Ac_6	NC_012483.1:578388	C>T	coding	-	GAG>GAA	E>E	syn	glutamate_formimidoyltransferase
Ac_6	NC_012483.1:2272157	C>T	coding	-	GTA>ATA	V>I	nonsyn	hypothetical_protein
Ac_7	NC_012483.1:1971981	G>C	noncoding					
Ac_8	NC_012483.1:1459340	A>T	coding	+	GAG>GTG	E>V	nonsyn	Beta-galactosidase_family_protein
Ac_9	NC_012483.1:1470241	G>A	coding	-	GCC>GCT	A>A	syn	non-specific_serine/threonine_protein_kinase
Ac_9	NC_012483.1:2533138	A>G	noncoding					
Ac_11	NC_012483.1:334628	G>T	noncoding					
Ac_11	NC_012483.1:3068321	C>A	coding	+	AGC>AGA	S>R	nonsyn	DNA_ligase,_ATP-dependent
Ac_12	NC_012483.1:3453815	T>C	coding	+	GTG>GCG	V>A	nonsyn	metal_ion_(Mn2+/Fe2+)_transporter_(Nramp)_family_transp
Ac_13	NC_012483.1:173318	G>C	coding	+	GTG>GTC	V>V	syn	acetyltransferase
Ac_13	NC_012483.1:3142757	C>T	noncoding					
Ac_13	NC_012483.1:3880351	C>T	coding	+	GCC>GTC	A>V	nonsyn	NodT_family_efflux_transporter_outer_membrane_lipoprotei
Ac_15	NC_012483.1:722614	T>C	coding	+	CAT>CAC	H>H	syn	phosphodiesterase
Ac_15	NC_012483.1:1629448	G>A	noncoding					
Ac_15	NC_012483.1:1786696	A>G	coding	-	AGT>AGC	S>S	syn	hypothetical_protein
Ac_15	NC_012483.1:3265382	A>G	coding	-	GAT>GAC	D>D	syn	TonB-dependent_receptor
Ac_16	NC_012483.1:3702647	C>T	coding	-	GCA>ACA	A>T	nonsyn	hypothetical_protein
Ac_17	NC_012483.1:2082223	C>T	noncoding					
Ac_17	NC_012483.1:4074092	G>A	coding	-	GCC>GTC	A>V	nonsyn	hypothetical_protein
Ac_18	NC_012483.1:2634113	C>T	coding	+	CAA>TAA	Q>*	nonsyn	outer_membrane_receptor_(OMR)_family_transporter
Ac_19	NC_012483.1:1610135	A>G	coding	-	TTT>TCT	F>S	nonsyn	hypothetical_protein
Ac_19	NC_012483.1:2864566	T>C	noncoding					
Ac_20	NC_012483.1:3787699	A>T	noncoding					
Ac_24	NC_012483.1:5120	G>A	coding	-	CCC>CCT	P>P	syn	hypothetical_protein
Ac_24	NC_012483.1:5126	A>G	coding	-	GCT>GCC	A>A	syn	hypothetical_protein
Ac_24	NC_012483.1:5129	G>A	coding	-	ATC>ATT	I>I	syn	hypothetical_protein
Ac_24	NC_012483.1:5132	G>A	coding	-	GCC>GCT	A>A	syn	hypothetical_protein
Ac_24	NC_012483.1:2625754	C>T	coding	-	GTG>GTA	V>V	syn	adenylosuccinate_synthetase
Ac_28	NC_012483.1:881692	G>A	noncoding					
Ac_30	NC_012483.1:431174	C>T	coding	+	AGC>AGT	S>S	syn	hypothetical_protein
Ac_31	NC_012483.1:2707729	C>T	coding	+	CGC>CGT	R>R	syn	FAD-binding/oxidase/4Fe-4S_binding_domain-containing_prot
Ac_34	NC_012483.1:3754475	C>T	coding	+	CGA>TGA	R>*	nonsyn	hypothetical_protein
Ac_35	NC_012483.1:71983	G>A	coding	+	GTG>ATG	V>M	nonsyn	alpha-galactosidase
Ac_35	NC_012483.1:1887722	T>A	coding	+	CTC>CAC	L>H	nonsyn	glycosyl_hydrolase,_family_39
Ac_36	NC_012483.1:2635372	G>A	coding	+	TGG>TGA	W>*	nonsyn	outer_membrane_receptor_(OMR)_family_transporter
Ac_42	NC_012483.1:4123155	A>T	coding	+	AAA>AAT	K>N	nonsyn	M20/M25/M40_family_peptidase
Ac_43	NC_012483.1:985254	C>A	noncoding					

Ac_45	NC_012483.1:515398	T>C	coding	+	ACT>ACC	T>T	syn	flippase_domain-containing_protein
Ac_47	NC_012483.1:2880630	G>T	coding	-	CTC>CTA	L>L	syn	hypothetical_protein
Ac_47	NC_012483.1:3764181	A>G	coding	-	TCT>CCT	S>P	nonsyn	helicase,_UvrD/REP_family
Ac_48	NC_012483.1:589956	G>A	coding	-	TTC>TTT	F>F	syn	crossover_junction_endodeoxyribonuclease_RuvC
Ac_50	NC_012483.1:834952	T>A	coding	+	CTG>CAG	L>Q	nonsyn	formate--tetrahydrofolate_ligase
Ac_50	NC_012483.1:2551383	G>A	noncoding					
Ac_52	NC_012483.1:798573	G>T	noncoding					
Ac_52	NC_012483.1:2824313	T>C	coding	+	ATC>ACC	I>T	nonsyn	cytochrome_c_oxidase_subunit_II
Ac_53	NC_012483.1:562220	G>A	coding	-	CAT>TAT	H>Y	nonsyn	tolB_protein
Ac_53	NC_012483.1:1070663	C>T	coding	-	CTG>CTA	L>L	syn	hypothetical_protein
Ac_53	NC_012483.1:4080731	C>T	coding	+	GCC>GCT	A>A	syn	hypothetical_protein
Ac_54	NC_012483.1:3527746	G>T	noncoding					
Ac_55	NC_012483.1:1760586	A>C	coding	-	TCG>GCG	S>A	nonsyn	hypothetical_protein
Ac_56	NC_012483.1:3260823	C>G	coding	+	CCC>CCG	P>P	syn	LacI_family_transcriptional_regulator
Ac_59	NC_012483.1:2986081	C>T	coding	-	CAG>CAA	Q>Q	syn	biotin--acetyl-CoA-carboxylase_ligase
Ac_59	NC_012483.1:3578274	C>G	coding	+	GCA>GGA	A>G	nonsyn	mandelate_racemase/muconate_lactonizing_protein
Ac_60	NC_012483.1:3213963	G>A	noncoding					
Ac_60	NC_012483.1:3213968	C>T	noncoding					
Ac_60	NC_012483.1:3213972	C>G	noncoding					
Ac_60	NC_012483.1:3213987	T>C	noncoding					
Ac_60	NC_012483.1:3214015	C>G	noncoding					
Ac_61	NC_012483.1:1859981	C>A	noncoding					
Ac_61	NC_012483.1:1859991	A>T	noncoding					
Ac_61	NC_012483.1:1859992	T>G	noncoding					
Ac_62	NC_012483.1:670329	G>A	coding	+	GGC>GAC	G>D	nonsyn	glyoxalase_family_protein
Ac_62	NC_012483.1:787294	T>C	coding	-	ACG>GCG	T>A	nonsyn	glycosyl_transferase
Ac_62	NC_012483.1:787300	C>A	coding	-	GGC>TGC	G>C	nonsyn	glycosyl_transferase
Ac_63	NC_012483.1:2635299	C>A	coding	+	TCA>TAA	S>*	nonsyn	outer_membrane_receptor_(OMR)_family_transporter
Ac_65	NC_012483.1:2632658	C>T	coding	+	CAG>TAG	Q>*	nonsyn	outer_membrane_receptor_(OMR)_family_transporter
Ac_65	NC_012483.1:3053098	G>A	coding	-	CGC>TGC	R>C	nonsyn	transglycosylase
Ac_66	NC_012483.1:1005612	T>A	coding	-	ACA>ACT	T>T	syn	lipoprotein
Ac_67	NC_012483.1:48001	T>C	coding	+	GGT>GGC	G>G	syn	hypothetical_protein
Ac_67	NC_012483.1:2872068	G>A	coding	-	TTC>TTT	F>F	syn	hypothetical_protein
Ac_69	NC_012483.1:1390473	C>T	noncoding					
Ac_69	NC_012483.1:4081306	C>T	coding	+	CCC>CTC	P>L	nonsyn	beta-lactamase
Ac_74	NC_012483.1:673065	G>A	coding	-	CGC>TGC	R>C	nonsyn	glycyl-tRNA_synthetase_subunit_beta
Ac_74	NC_012483.1:2983187	A>T	coding	-	CTG>CAG	L>Q	nonsyn	23S_rRNA_(uracil-5-)-methyltransferase
Ac_75	NC_012483.1:1663106	G>A	coding	-	CTC>TTC	L>F	nonsyn	Ig_domain-containing_protein
Ac_76	NC_012483.1:2546888	T>C	coding	+	CTG>CCG	L>P	nonsyn	pectin_acetylesterase
Ac_76	NC_012483.1:2747650	A>G	coding	-	CTG>CCG	L>P	nonsyn	hypothetical_protein
Ac_77	NC_012483.1:2514716	C>G	coding	+	CGT>GGT	R>G	nonsyn	hypothetical_protein
Ac_78	NC_012483.1:2635156	T>G	coding	+	TAT>TAG	Y>*	nonsyn	outer_membrane_receptor_(OMR)_family_transporter
Ac_78	NC_012483.1:3872314	C>T	noncoding					
Ac_79	NC_012483.1:1914804	G>A	noncoding					
Ac_80	NC_012483.1:1946783	T>C	coding	-	AAT>AGT	N>S	nonsyn	DNA-binding_protein_HU_1

Ac_80	NC_012483.1:3068203	C>A	coding	+	CCT>CAT	P>H	nonsyn	DNA_ligase,_ATP-dependent
