# Supplementary material for: Low base‐substitution mutation rate and predominance of insertion‐deletion events in the acidophilic bacterium Acidobacterium capsulatum
Source: Ecol Evol. 2021 Dec 17;11(24):17609–14. doi: 10.1002/ece3.8429 (PMC8717266; doi:10.1002/ece3.8429)
Supplement: Supplementary file 4 — Table S4 [file ECE3-11-17609-s002.pdf]

Suppl. Table 4. Indel details

MA Line	Chromosome	Position	Reference	Indels	Genic region	Gene	SSR
Ac_1	NC_012483.1	2634017	C	CA	coding	outer membrane receptor (OMR) family transporter	-
Ac_6	NC_012483.1	4364	C	CGTAAATA	coding	hypothetical protein	-
Ac_8	NC_012483.1	2634972	T	TC	coding	outer membrane receptor (OMR) family transporter	5c
Ac_9	NC_012483.1	3206362	AG	A	noncoding	-	-
Ac_11	NC_012483.1	401405	GC	G	coding	hypothetical protein	6c
Ac_12	NC_012483.1	3957	AG	A	coding	hypothetical protein	-
Ac_13	NC_012483.1	3975241	G	GA	noncoding	-	7a
Ac_19	NC_012483.1	2800140	GCTGCGCA	C	coding	alpha glucuronidase	-
Ac_20	NC_012483.1	1504683	C	CG	coding	Tat pathway signal sequence domain-containing protein	5g
Ac_23	NC_012483.1	2484163	CAGCAGCGA	A	coding	macrolide ABC transporter permease	-
Ac_34	NC_012483.1	2909647	A	AG	coding	trehalase	4g
Ac_36	NC_012483.1	1993159	T	AAGCGGT	coding	ribonuclease, Rne/Rng family, insertion	-
Ac_45	NC_012483.1	2633132	AT	A	coding	outer membrane receptor (OMR) family transporter	5t
Ac_46	NC_012483.1	1593749	GCGCCCTG	T	coding	hydrophobe/amphiphile efflux-1 family transporter	-
Ac_47	NC_012483.1	2899727	TCG	T	coding	sensor histidine kinase	5cg
Ac_49	NC_012483.1	3003750	C	CTGAGGA	noncoding	-	-
Ac_60	NC_012483.1	3213967	T	TG	noncoding	-	-
Ac_61	NC_012483.1	1938376	C	AGTCCTCA	noncoding	-	-
Ac_62	NC_012483.1	787297	AGC	A	coding	glycosyl transferase	-
Ac_62	NC_012483.1	1611523	ACTGGCTGA	A	noncoding	-	-
Ac_67	NC_012483.1	758491	T	TG	noncoding	-	7g
Ac_67	NC_012483.1	2021344	CGACCCGCT	C	noncoding	-	3gacccgct
Ac_68	NC_012483.1	482087	GCGGAATG	C	coding	ortho-chlorophenol reductive dehalogenase	-
Ac_68	NC_012483.1	2013749	GGAA	G	coding	hypothetical protein	8gaa
Ac_69	NC_012483.1	1548379	A	AG	coding	S53 family peptidase	-
Ac_74	NC_012483.1	2191208	C	CTAGCTTT	noncoding	-	-
Ac_75	NC_012483.1	3795730	CTCCCAATA	G	noncoding	-	-
Ac_78	NC_012483.1	3438360	A	AG	noncoding	-	4g
Ac_80	NC_012483.1	288000	AGTTTGCCG	T	coding	flagellar basal-body rod protein FlgC	-
Ac_80	NC_012483.1	2687342	G	AGCGAATG	coding	L-serine ammonia-lyase	-
Ac_80	NC_012483.1	2899981	TG	T	coding	sensor histidine kinase	4g
